# Deep sequencing combined with microarray hybridization to identify novel and conserved microRNAs during somatic embryogenesis of hybrid yellow-poplar (*Liriodendron chinense* (Hemsl.) Sarg. X *L. tulipifera* Linn.)

**DOI:** 10.1186/1753-6561-5-S7-P74

**Published:** 2011-09-13

**Authors:** Li Tingting, Chen Jinhui, Shi Jisen, Xu Jin

**Affiliations:** 1 The key laboratory of forest genetics and gene engineering of the ministry of education of China Nanjing Forestry University Nanjing 210019, China

## Background

Hybrid yellow-poplar was a man-made cross between *Liriodendron tulipifera* Linn.× *L. chinense*(Hemsl.) Sarg.. Heterosis was strong both on growth and adaptation, no matter what, the crosses or the reciprocals. During the last 12 years, somatic embryogenesis systems were successfully established in our lab, in order to meet the needs of planting materials for expanding plantations in Southern China. Synchronous development of high quality embryos was always required both for the study of embryo development, and the supply of commercial emblings. The functions of plant microRNAs (miRNAs) in post-transcriptional gene regulation by targeting mRNAs for degradation, or the translational repression by hybridizing to complementary sequences within target mRNA molecules were well reported [1]. One of the major roles of miRNAs in regulating plant development has been obtained from a number of studiesbut molecular mechanisms regulating the development of embryos are not well understood, especially during the early stages of somatic embryos [2]. Here we present some results on the discovery of novel and conserved miRNAs during somatic embryogenesis of hybrid yellow-poplar using Illumina sequencing and miRNA microfluidic chip in order to understand how the embryogenic somatic mass is developed into embryos and plants.

## Materials and methods

The pre-embryo mass (PEM) and different stages of somatic embryos (SE) (Figure 1) were collected sequentially from the hybrid yellow-poplar somatic embryogenesis system. All the callus and embryo tissues were staged by microscope and immediately forzen in liquid nitrogen and stored at -80C until used. Total RNA was isolated from different developmental stages of somatic embryogenesis using *Total RNA Purification Kit* (Norgen Biotek Corporation, Canada). All the RNA samples from different somatic embryo tissues were mixed equally to form a single RNA pool. Following the instruction of LC Science, small RNA sample was prepared and then sequenced using Illumina Technology. Processing and analysis of raw data was done following Sunkar et al [3,4]. Conserved and candidate novel miRNAs in hybrid yellow-poplar were identified . A mixed small RNA microarray hybrid was used to validate the sequencing result and detect new miRNAs in hybrid yellow-poplar; 299 sequences detected by sequencing and 1,450 plant miRNAs sequences in miRBase 15.0 were ordered to serve as probes. A well-developed stem-loop strategy [5]and SYBR Green master mix were used to perform real time PCR to validate the miRNAs expressed in both sequencing and microarray analysis. The potential targets of miRNAs were predicted using the psRNATarget program (http://bioinfo3.noble.org/psRNATarget/) with default parameters. The biology and molecular functions of these predicted targets were categorized by the Gene Ontology program (http://www.Geneontology.org/).

## Results

A total of 17,214,153 reads representing 7,421,623 distinct sequences were obtained from a short RNA library generated from small RNA extracted from all stages of somatic embryogenesis tissues. Hybrid yellow-poplar has a complex small RNA population and the length of small RNAs varied. The 24-nt and 21-nt are the predominant length for the majority of the small RNAs. Combining deep sequencing and bioinformatics analysisthere were 82 sequences, with perfectly matched known miRNAs from 32 conserved miRNAs families and 273 species-specific candidate miRNAs whose precursors were generated from *Liriodendron tulipifera* ESTs and form good hairpin structure. Among these miRNAs, both miR156 and miR166 which have been reported to be important during plant early embryonic patterning, had a large family members expressed in this process. Microarray hybridization and qRT-PCR technology were used to demonstrate that most conserved and species-specific miRNAs expressed in hybrid yellow-poplar. In addition, another set of 149 miRNAs included in 29 conserved families was found, which were not discovered by deep sequencing analysis but have obvious detectable signals in the microarray. Potential targets of biological processes and molecular functions of these miRNAs were predicted by blasting with *Arabidopsis thaliana* and analyzing with Gene Ontology. These predicted target genes participate in many metabolic and biological processes such as transport, signal transduction, response to stress, cell organization and many other cellular or developmental processes.

## Conclusions

By combining Illumina deep sequencing with microarray hybridization we discovered 231 miRNAs from 61 conserved miRNA families and 273 species-specific candidate miRNAs. The potential miRNA target functions related to various biological and metabolic processes. These results indicate that microRNAs play wide-ranging and important roles during all stages of somatic embryogenesis in hybrid yellow–poplar. Additional experiments on the functional verification of interesting candidate miRNAs are now under consideration.
